# Glucose, Nitrogen, and Phosphate Repletion in *Saccharomyces cerevisiae*: Common Transcriptional Responses to Different Nutrient Signals

**DOI:** 10.1534/g3.112.002808

**Published:** 2012-09-01

**Authors:** Michael K. Conway, Douglas Grunwald, Warren Heideman

**Affiliations:** Pharmaceutical Sciences, School of Pharmacy, University of Wisconsin, Madison, Wisconsin 53705

**Keywords:** mitogenesis, glucose, yeast, transcription, RRPE, glucose, nitrogen, phosphate, protein kinase A, TOR, cAMP

## Abstract

*Saccharomyces cerevisiae* are able to control growth in response to changes in nutrient availability. The limitation for single macronutrients, including nitrogen (N) and phosphate (P), produces stable arrest in G1/G0. Restoration of the limiting nutrient quickly restores growth. It has been shown that glucose (G) depletion/repletion very rapidly alters the levels of more than 2000 transcripts by at least 2-fold, a large portion of which are involved with either protein production in growth or stress responses in starvation. Although the signals generated by G, N, and P are thought to be quite distinct, we tested the hypothesis that depletion and repletion of any of these three nutrients would affect a common core set of genes as part of a generalized response to conditions that promote growth and quiescence. We found that the response to depletion of G, N, or P produced similar quiescent states with largely similar transcriptomes. As we predicted, repletion of each of the nutrients G, N, or P induced a large (501) common core set of genes and repressed a large (616) common gene set. Each nutrient also produced nutrient-specific transcript changes. The transcriptional responses to each of the three nutrients depended on cAMP and, to a lesser extent, the TOR pathway. All three nutrients stimulated cAMP production within minutes of repletion, and artificially increasing cAMP levels was sufficient to replicate much of the core transcriptional response. The recently identified transceptors Gap1, Mep1, Mep2, and Mep3, as well as Pho84, all played some role in the core transcriptional responses to N or P. As expected, we found some evidence of cross talk between nutrient signals, yet each nutrient sends distinct signals.

Yeast starved for macronutrients, such as glucose (G), nitrogen (N), or phosphorous (P), arrest growth and cell division and become quiescent, with cell wall thickening, reduced transcription and translation, and increased stress tolerance (Gray *et al.* 2004; Rowley *et al.* 1993). Upon nutrient repletion, yeast immediately return to growth and division (Unger and Hartwell 1976).

Glucose addition to starved, quiescent yeast rapidly alters the expression of more than a third of the yeast genome by at least 2-fold (Martinez *et al.* 2004; Radonjic *et al.* 2005; Slattery and Heideman 2007; Wang *et al.* 2004). Genes needed for ribosome biogenesis (RiBi), ribosomal proteins (RP) translation, mass accumulation, and cell division are induced (Jorgensen *et al.* 2004). In contrast, environmental stress response (ESR) (Gasch *et al.* 2000), gluconeogenic, respirative, and alternative metabolism genes are repressed.

This large-scale change depends on the Gpa2 G-protein associated with the Gpr1 glucose receptor (Santangelo 2006; Wang *et al.* 2004). The response is also largely dependent on cAMP production, as well as a functional TOR pathway (Slattery *et al.* 2008). Glucose produces this massive rearrangement of the transcriptome, even in cells lacking the ability to take up and metabolize glucose (Slattery *et al.* 2008). These results point to a cell surface receptor-mediated response.

Most study of nutrient sensing in *S. cerevisiae* has focused on specific metabolic challenges, such as glucose repression or regulation of amino acid synthesis. Thus, we have limited knowledge of how these different nutrients cause cells to return to growth (Forsberg and Ljungdahl 2001; Magasanik and Kaiser 2002; Wykoff and O’Shea 2001; Zaman *et al.* 2008).

Work from Thevelein *et al.* (2005) has shown a role in trehalase activation for cell surface N and P sensors that is largely dependent on PKA. Because these proteins needed for trehalase stimulation also serve as nutrient transporters, they have been termed transceptors (Donaton *et al.* 2003; Popova *et al.* 2010; Van Zeebroeck *et al.* 2009). One such transceptor is the Gap1 amino acid transporter (Jauniaux and Grenson 1990; Magasanik and Kaiser 2002). Trehalase activation by amino acids is Gap1-dependent. A similar finding was made in studying the role of the Pho84 phosphate transporter. Phosphate activation of trehalase activation is Pho84-dependent (Popova *et al.* 2010). Finally, the Mep family of ammonium transporters are important for ammonium stimulation of trehalase activity (Van Nuland *et al.* 2006).

Because the states of quiescence and growth appear have requirements that are the same regardless of the missing nutrient, we hypothesized that N, P, and G depletion and repletion would control large common gene sets needed for growth and quiescence. In this article, we confirm this hypothesis and show that all three nutrients produce responses that are similar in appearance and share common mechanisms. While the initial signals appear to come from different receptors, including Gpr1, Gap1, Pho84, and Mep proteins, the nutrient signals produce a response that is largely cAMP-dependent. Furthermore, all three nutrients elevate cAMP levels when repleted. These results indicate that different nutrient signals converge to control common states of quiescence and growth.

## Materials and Methods

### Yeast strains and growth media

S288C (*MATα SUC2gal2 mal mel flo1flo8-1 hap1hobio1bio6*) was used for glucose, nitrogen, and phosphate experiments. TC41-1 (*MATα leu2-3 leu2-112 trp1-1 his3-532 his4cyr1*::*URA3cam*) and the isogenic *CYR1*+ wild-type HR125 were used for *PKA* and *TOR* nutrient repletion experiments (Heideman *et al.* 1990). BY4742 (*MATα his3Δ1 leu2Δ0 lys2Δ0 ura3Δ0*) was used for transceptor work as wild-type and *gap1Δ* and *pho84Δ* mutants made in BY4742 were from the yeast knockout collection obtained from Open Biosystems, with either gene deleted by KanMX. Deletions were confirmed by PCR.

### Growth conditions

Cells were grown in YPD (1% yeast extract, 2% peptone, 2% glucose, Sunrise Chemicals) or synthetic medium (SD) with 2% glucose containing 6.7 g/l yeast nitrogen base (US Biological) supplemented with adenine, uracil, and amino acids. Cells were cultured at 30° with shaking. Nitrogen deprivation medium (SD-N) consisted of SD made up with nitrogen-free Yeast Nitrogen Base (Difco) and without amino acids or uracil. Phosphate deprivation medium (SD-P) consisted of SD made with yeast nitrogen base in which potassium chloride was substituted for potassium phosphate. Rapamycin (10 µg/ml stock in ethanol, LC Laboratories) and cAMP (1M stock in water, pH 7, Sigma) treatments were as described previously (Newcomb *et al.* 2003; Russell *et al.* 1993; Slattery *et al.* 2008). When added, cAMP was used at 1 mM and rapamycin at 200 nM.

### Nutrient depletion

Glucose depletion was as previously described (Slattery and Heideman 2007). Cells were grown in SD medium for 48–72 hr until they had arrested as a quiescent G1 phase population. Nitrogen starvation was achieved by inoculating cells from an overnight SD culture into SD-N at a density of 0.4 OD_660_. When growth stopped (24 hr, approximately 1.5 OD_660_), cells were transferred to a fresh volume of SD-N to a density of 0.5 OD_660_. These cells were incubated an additional 24 hr and generally reached a density of OD_660_ 0.75–1. Finally, this culture was resuspended in fresh SD-N at OD_660_ of 1.0 and incubated for 12–24 hr. Depletion was confirmed by determining that cells would not proliferate in fresh SD-N but would grow in SD. Phosphate depletion was carried out in the same manner, except that phosphate depletion medium was used. In both cases, nutrient repletion was accomplished by pelleting in a Beckman J6 centrifuge at 30° at 2500 rpm for 5 min and resuspension in an equivalent volume of fresh SD medium.

For dual nutrient depletion experiments, cultures were grown in N- or P-free medium until they ceased dividing and were then transferred to medium also lacking G and incubated for an additional 48 hr to deplete any glucose remaining in the medium. This produced GN- and GP-depleted cells; we confirmed that repletion of only a single nutrient did not produce growth (data not shown).

The *pho84Δ* cells were starved for P as described above and challenged with KH_2_PO_4_ or Gly3P (both 10 mM). The Gap1 cells were nitrogen depleted as described above and repleted with SD-N with 10 mM L-citrulline added. The MEP-deletion strains were N-depleted as described above and repleted by addition of SD-N plus 10 mM ammonium sulfate.

The *cyr1*Δ strain TC41 was nutrient depleted using the techniques previously described (Slattery and Heideman 2007), in which the nutrient depletion followed the schedule described above except that during nutrient the first 24 hr of depletion the cells were cultured with 1 mM cAMP and 0.5× auxotrophic supplements to be certain that the cells could remain growing enough to deplete the missing macronutrient. This was followed by 24 hr in depletion medium with 1 mM cAMP and by an additional 24 hr with no cAMP. This procedure was used to avoid halting growth and metabolism prematurely by cAMP withdrawal before true nutrient depletion had occurred.

### cAMP measurements

Twenty-five optical density (OD) units of cells were harvested into ice-cold TCA to a final concentration of 5% and vortexed briefly to mix. The lysates were neutralized with NaHCO_3_ to pH ∼6.5–7 and snap-frozen on liquid nitrogen. Immediately prior to assay, samples were thawed on ice and centrifuged at 4° for 1 min, and then 20 µL of supernatant was used in the R&D Systems cAMP Parameter Assay Kit (KGE002B) as indicated by the manufacturer.

### RNA isolation and microarray hybridization

Samples of cells were collected in independent experiments to produce true biological replicates. For these experiments, cultures from independent colonies on the same plate were started and grown in separate flasks to nutrient limitation as described above. In some cases, duplicate cultures were grown on separate days, and in others, the cultures were grown in the same shaker started on the same day. Samples were kept separate, and the results from the duplicates are shown.

For each hybridization, 10 ODU cells were collected and pelleted at 5000 RPM in a Beckman J6 centrifuge for 2 min at 30°. Then the supernatant was removed and the pellets were frozen with liquid nitrogen.

RNA was isolated using MasterPure Yeast RNA Purification Kits (Epicentre Technologies), and the quality was assayed by gel electrophoresis. cRNA synthesis was carried out using the GeneChip Expression 3′ Amplification One-Cycle Target Labeling and Control Reagents kit from Affymetrix following the manufacturer’s instructions. cRNA samples were hybridized to GeneChip Yeast Genome 2.0 Arrays for 16 hr. Arrays were washed, stained, and scanned according to the manufacture’s recommendation. Affymetrix .CEL files were RMA normalized with R and the Bioconductor Suite (Gentleman *et al.* 2004). Data analysis was performed within TIGR Multiexperiment Viewer, v4.5.1 (Saeed *et al.* 2003; 2006), in-house Perl scripting, R, and Bioconductor.

Genes that were differentially expressed between the fed and starved states from each nutrient condition were selected using the following criteria: a *P* value less than 0.05; a false discovery rate less than 0.01 (q-value); and a 2-fold change in expression. The individual nutrient lists were compared to identify the genes in common, as well as those unique to each combination, resulting in the Venn diagrams shown in Figures 3 and 4 and supporting information, Table S1 and Table S2.

GO enrichment analysis was performed through FUNSPEC tool (Robinson *et al.* 2002) at http://funspec.med.utoronto.ca/. Under- and overrepresented DNA motifs were identified using the RSAT online motif discovery tool at http://rsat.ulb.ac.be/ (van Helden 2003), where oligomer length was scanned between 4 and 8 bases and the best-consensus sequence scores were collected. Transcription factor target and motif enrichment significance were calculated using a hypergeometric distribution test.

## Results

### Different nutrient limitations produce a similar transcriptome

Starvation for glucose (G), nitrogen (N), or phosphate (P) each produces a morphologically similar G1 arrested population (Gasch *et al.* 2000; Giots *et al.* 2003; Gray *et al.* 2004; Johnston *et al.* 1977; Mazon 1978). This leads to the idea that while the signals from these nutrients are distinct, they converge in some way to control quiescence and growth.

**Figure 1:**
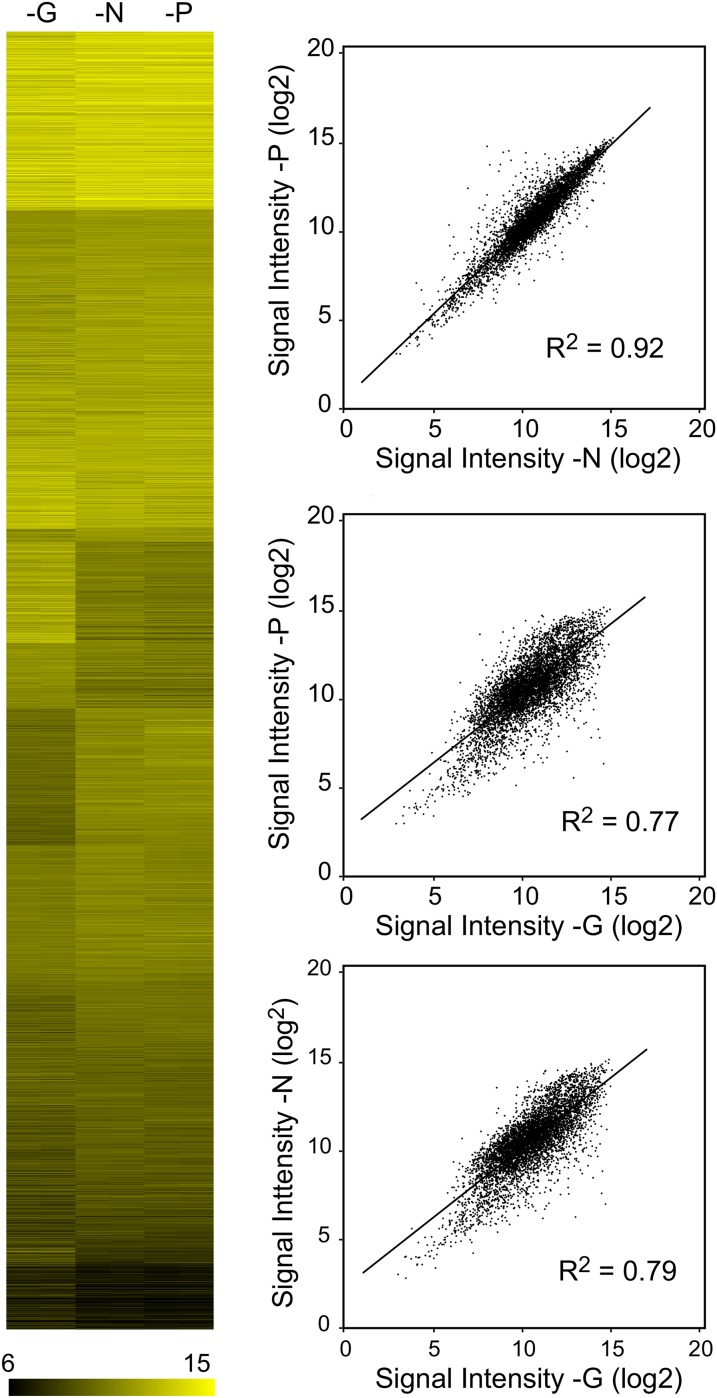
Comparing transcript levels in G-, N-, or P-depleted cells. Wild-type (S288C) cultures were transferred into S medium lacking G, N, or P and were cultured until growth was arrested, and then samples were collected for Affymetrix microarray analysis as described in *Materials and Methods*. (A) Heat map plotting normalized log2-transformed hybridization intensity data for G-, N-, and P-limited samples arranged by k-means clustering. Independent biological replicate samples are shown as side-by-side columns; the values are so similar between replicates that these columns cannot be readily distinguished by eye in most cases. (B) For each transcript, average intensity data from (A) is plotted such that the expression level in one nutrient condition is on the X-axis, and intensity value in another condition is on the Y-axis. G *vs.* N starvation yielded a Pearson correlation of 0.79; G *vs.* P starvation a correlation of 0.77; and N compared with P, a correlation of 0.92.

We first compared transcripts in yeast starved for G, N, or P. Prototrophic S288C cells were transferred from complete medium to synthetic medium lacking G, N, or P and incubated until they had arrested growth as described in *Materials and Methods*. Total RNA was isolated and used to probe Affymetrix microarrays. The experiments were conducted using duplicate yeast cultures started from separate colonies. Paired biological replicate signal intensities for each mRNA for each starvation condition are shown as a heat map (Figure 1A). In this arrangement, genes at the top of the map with the brightest color have the highest hybridization signals, whereas those at the bottom had the lowest. Replicate experiments are shown side by side, but in most cases, the replicate values were so similar that they cannot be visually distinguished.

Qualitatively, the heat map in Figure 1A indicates that all three nutrient limitations produce regions of strong similarity in transcript abundance pattern; with the G-depleted samples somewhat distinct from the N- and P-depleted samples. We calculated Pearson correlation coefficients and produced linear regression plots to compare the patterns of transcript abundances produced by each type of depletion (Figure 1B). Each transcript is plotted as a dot positioned with its abundance in one nutrient condition plotted on the Y-axis, and abundance in the other nutrient condition on the X-axis. Overall, there was high correlation between the three starvation states (Chua *et al.* 2006; Grigull *et al.* 2004), but as noted, the responses to N and P limitation were more similar to each other than either was to the response produced by G limitation. Comparison of the N and P starvation responses produced a correlation of 0.93, whereas comparison of G limitation to N and P starvation yielded Pearson correlations of 0.79 and 0.77 respectively.

In general, these results indicate that the quiescent states produced by the separate nutrient limitations are quite similar in terms of global gene expression, a conclusion recently reported by Klosinska *et al.* (2011). Although we used an S288C strain and Broach and co-workers in that study used a W303 derivative, both experiments yielded similar results. Comparing our single time point values with the published results, we found the highest correlations to their 5760 (96 hr) time point samples, with Pearson correlations of 0.78, 0.75, and 0.64 between the G-, N-, and P-depletion results, respectively.

### Nutrient repletion triggers massive changes in transcript abundance

The cellular pathways for sensing G, N, or P are thought to be quite distinct (Santangelo 2006; Schneper *et al.* 2004; Zaman *et al.* 2008). Yet, repletion of each of these nutrients causes growth, protein synthesis, and reversal of quiescence. We hypothesized that repletion of each nutrient would produce a set of transcriptional changes overlapping those produced by G (Radonjic *et al.* 2005; Slattery and Heideman 2007; Wang *et al.* 2004).

**Figure 2:**
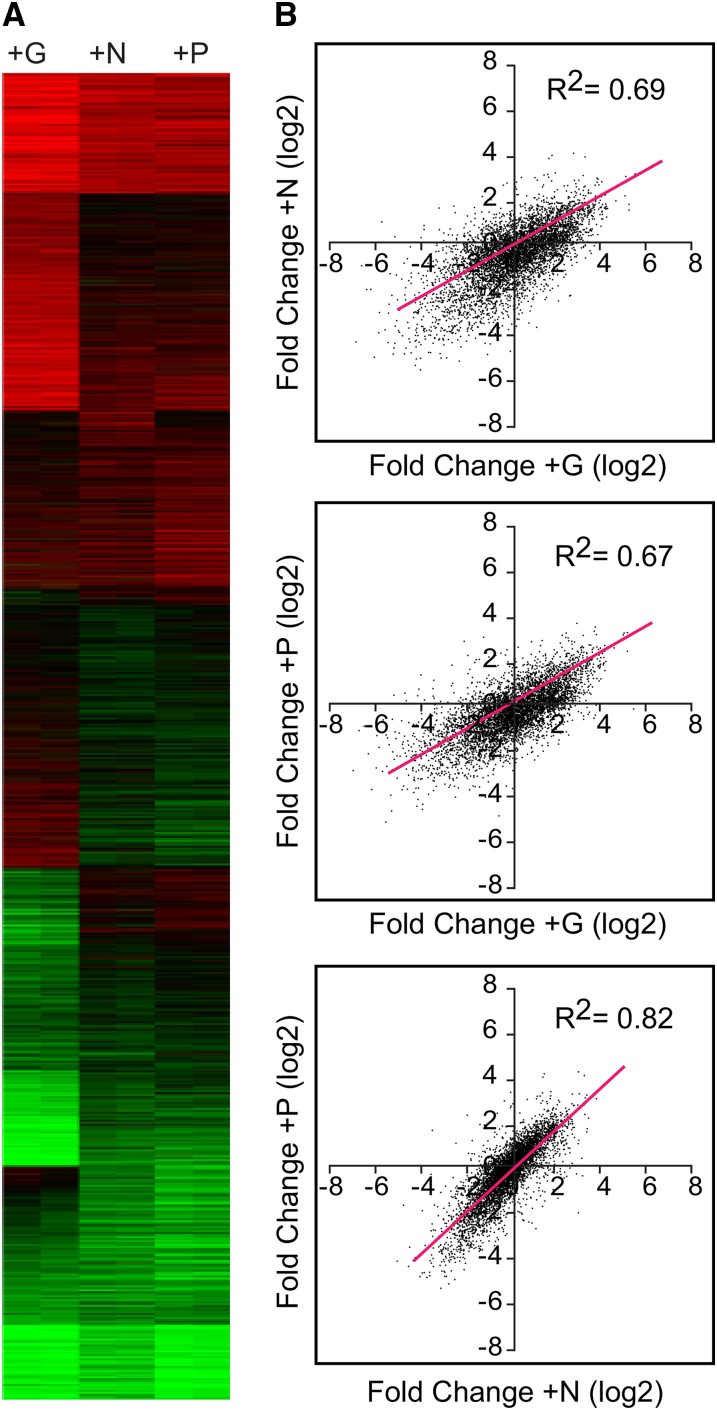
Large-scale transcriptional responses to G, N, and P repletion. Wild-type (S288C) cells were starved for G, N, or P until growth was arrested as in Figure 1, and then the missing nutrient was repleted as described in *Materials and Methods*. Samples for microarray analysis were taken at the nutrient-limited state and 60 min after repletion of each missing nutrient. (A) Heat map showing the log2 fold change for each gene, comparing the nutrient repleted with the initial quiescent samples. Independent biological replicate samples are shown as side-by-side columns, and the transcripts are arranged by k-means clustering. Red indicates increased expression; green indicates reduced. (B) For each transcript, fold change data from (A) is averaged and plotted such that the fold change in response to one nutrient condition is on the X-axis, and the fold change in response to another nutrient is plotted on the Y-axis. The red diagonal line indicates the linear regression: G compared with N repletion yielded a Pearson correlation of 0.69, whereas compared with P, yielded 0.67. N *vs.* P repletion had a correlation of 0.83.

To test this, we added back the limiting nutrient to quiescent G-, N- or P-depleted S288C cells and collected samples for microarray analysis at 60 min. By 90 min, cells had begun to produce buds, indicating a return to growth (not shown). Figure 2A shows the changes in gene expression produced by each nutrient, expressed as fold change (log2) relative to quiescent samples. Red indicates increased expression, and green indicates repression caused by nutrient repletion.

Large groups of genes were upregulated by all three nutrient repletions, as revealed by clusters toward the top of the figure, whereas other groups, shown toward the bottom, were downregulated by all three repletions. Other gene sets were nutrient-specific.

As with the data in the first figure, we used dot plots to compare the fold change induced by one nutrient with the changes induced by another (Figure 2B). As before, the similarities between the responses were reflected in the correlation coefficients, and the N- and P-repletion responses were more similar to each other than they were to the G response. N and P repletion produced responses with a correlation of 0.82, whereas the response to N repletion produced a correlation coefficient of 0.69 compared with the response to G. Comparison of the responses to G and P repletion produced a correlation of 0.67.

### Growth genes are upregulated by all three nutrients

To identify a core set of genes induced in response to all three nutrients, we selected sets of genes that were induced by each of the nutrient repletions. These sets were made up of transcripts that met the following criteria: *P* < 0.05, a false discovery rate (q-value) less than 0.01, and at least a 2-fold induction by the nutrient repletion (log2 ratio greater than or equal to 1).

**Figure 3:**
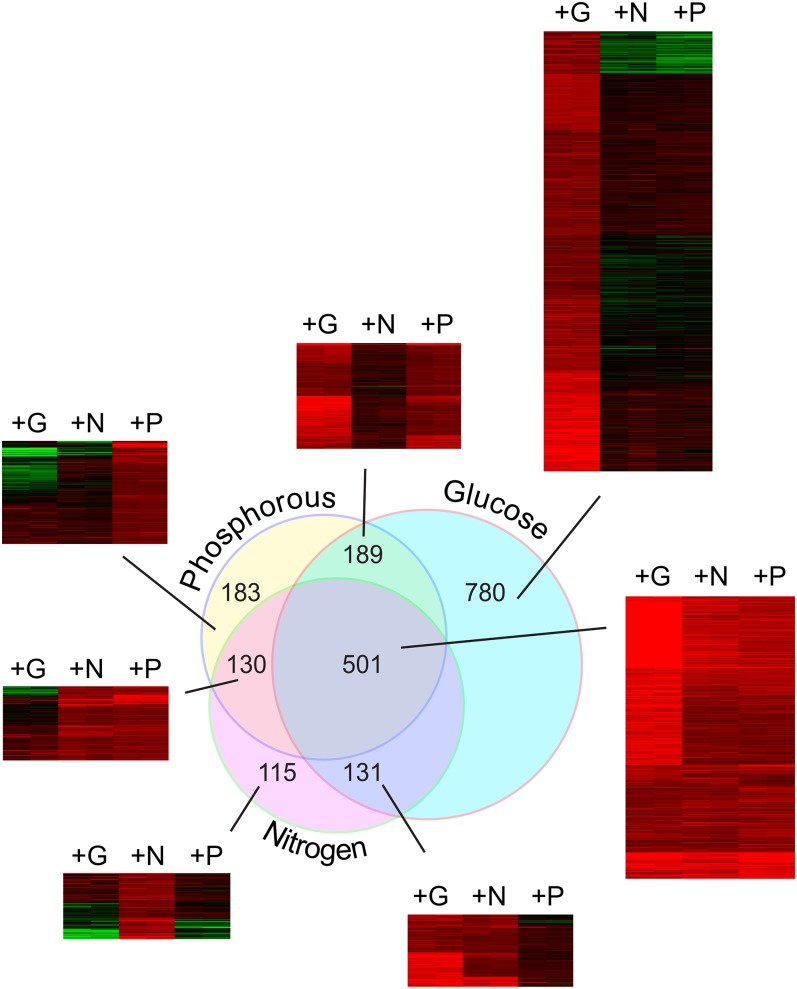
Genes induced by G, N, and P repletion. Microarray data from Figure 2 was used to identify genes induced at least 2-fold by G, N, or P repletion using cutoffs described in *Materials and Methods*. The Venn diagram shows the intersections between the sets of genes induced at least 2-fold by each nutrient. This intersection of three sets produced seven different groups, and a heat map of fold-change responses is shown for each of these seven sets. Independent biological replicate samples are shown as side-by-side columns, and the transcripts are arranged by k-means clustering. The number of individual transcripts in each set is shown in the figure, and the transcripts are listed for each set in Table S1.

This arbitrary cutoff yielded the following gene sets: 1601 increased by glucose; 877 increased by nitrogen; and 1003 increased by phosphate. Of the genes upregulated by both nitrogen and phosphate, 83% (*P* < 2.139 × 10^−257^) were also upregulated by glucose, producing an overlapping set of 501 genes induced by each of the three nutrients (Figure 3).

This common set of 501 genes was greatly enriched for protein synthesis genes. GO functional classification showed significant enrichment for rRNA processing (<1e^−14^), tRNA processing (<1e^−14^), ribosome biogenesis (<1e^−14^), rRNA synthesis (<1.84e^−12^), and related growth functions (Table 1). This set was also significantly enriched for genes mapping to the nucleolus (<1e^−14^), a key site for ribosome development. Of the 236 recognized RiBi transcripts we measured, 160 (67%, *P* < 1.145e^−117^) were found in this common cluster (Jorgensen *et al.* 2004). G, N, and P repletions all induced ribosomal protein (RP) transcripts; however, the fold induction by G averaged well above the 2-fold cutoff, while for most RP genes, induction by N or P fell slightly below this cutoff (Table S1).

**Table 1:** Transcription factor target and binding motif enrichment, gene ontology enrichment, and overrepresented promoter motifs found in the induced Venn diagram genes

Induced Cluster	TF Bound	Enriched TF Motifs	Enriched GO Terms	Overrepresented Motifs
G, N, P	ABF1, YOX1, REB1, LEU3^*^, CHA4^*^, RPH1^*^, RIM101^*^, PT23^**^, DAL80^**^, ARR1^**^	CHA4, STB2, REB1, ABF1, AZF1, PHO2, XBP1, MOT3^*^, DAL80^*^, YER051W^*^	rRNA processing (11.04.01),	aagtgaaaaatttca
			tRNA modification (11.06.02),	aagctcatcgcat
			Ribosome biogenesis (12.01),	aagtgaaaaaaat
			RNA binding (16.03.03),	aaaagaaaaaaat
			rRNA synthesis (11.02.01),	gagatgagatga
			Translation initiation (12.04.01)	aagctcatcgcaaa
G only	FHL1, RAP1,	SFP1, FHL1, RAP1, SWI6, MBP1, STB1, SWI4, FKH2, YRR1, STB2	Ribosomal proteins (12.01.01)	aacgcgtt, attgaaaaac
	MBP1, SFP1,		ER to Golgi transport (20.09.07.03)	aacgcgaaaaac
	SWI6, SWI4,		Triterpene metabolism (01.06.06.11)	aaaggt
	FKH2, RFX1,		Chromosome segregation/division 10.03.04.05)	aaagaaa
	MCM1^*^, ASH1^*^		Glycosylation, deglycosylation 14.07.02.02)	agacgcgtt
			Translation elongation (12.04.02)	
N only	GCN4, BAS1, MET32, RTG3, MET4, CBF1, STP4, GAT3, STB5^*^, PUT3^*^	BAS1, ARG81, ARG80, GCN4, MET32, MET4, RTG3, CBF1, SFL1, AFT2	Amino acid metabolism (GO:0006519),	agagtcat
			Nitrogen metabolic process (GO:0006807),	ccacagt
			Purine nucleotide/nucleoside/ nucleobase anabolism (01.03.01.03),	
			Sulfate assimilation (01.02.03.01),	
			NAD/NADP binding (16.21.07),	
			Aminoadipic acid pathway (01.01.06.06.01.03)	
P only	INO4, INO2, STP1, ARG81, RPN4^*^, HAP3^*^, ARG80^*^, ABF1^*^, STB5^*^, PDR1^*^	ABF1, SPT23, CBF1, SKN7, SWI5, AZF1, NRG1, RPN4, YAP5, MSN4^*^	Transcriptional control (11.02.03.04),	acggcg
			DNA binding (16.03.01),	
			DNA conformation modification (*e.g.* chromatin) (10.01.09.05),	
			Transcription repression (11.02.03.04.03),	
			General transcription activities (11.02.03.01)	
G, N	GCN4, STB5, STB1, TYE7^*^, RGT1^*^, GLN3^*^, INO4^*^, SWI6^*^, STB2^*^, OPI1^*^	ARR1, HAP2, RAP1, SPT2, YAP3, CHA4, HAP4, GCN4, FKH2, YHP1^*^	Biosynthesis of tryptophan (01.01.09.06.01),	tgaaaa
			Pentose-phosphate pathway (02.07),	
			Biosynthesis of histidine (01.01.09.07.01),	
			N-directed glycosylation, deglycosylation (14.07.02.02),	
			Purine nucleotide/nucleoside/nucleobase anabolism (01.03.01.03),	
			Aminoadipic acid pathway (01.01.06.06.01.03)	
G, P	STE12, DIG1, FKH2, SWI4, FKH1, SWI6, STB1, HAP5^*^, MATA1^*^, CHA4^*^	RLM1, YER051W, SFL1, SWI4, STB1, SWI5, GLN3^*^, MBP1^*^, THI2^*^, STB4^*^	Sexual reproduction (GO:0019953),	attttcgaaaat
			Pheromone response, mating-type determination (34.11.03.07),	attttcgcgaaaat
			Cytoskeleton/structural (42.04)	gcgaaat
			Ori recognition and priming complex formation (10.01.03.03),	
			Chitin anabolism (01.05.03.03.04),	
			G2/M transition of mitotic cell cycle (10.03.01.01.09)	
N, P	ARG81, YAP7, AZF1, NRG1, ARG80, SPT23^*^, MET32^*^, GZF3^*^, MET31^*^, INO4^*^	CRZ1, XBP1, RTG3, SKO1, RGT1, CHA4, GAL80, RCS1^*^, STP1^*^, RDS1^*^	Ribosome biogenesis (GO:0042254),	aaaaaaa
			rRNA processing (GO:0006364)(GO:0000447)(GO:0000472)	cgatgag
			Ribosomal small subunit biogenesis (GO:0042274),	
			Endonucleolytic cleavage in 5′-ETS of pre-rRNA	

Each of the seven gene clusters identified in Figure 3A was searched for transcription factor target gene enrichment, transcription factor motifs in gene promoter regions, gene ontology (GO) enrichment, and overrepresented promoter motifs. The column TF Bound indicates transcription factors whose targets were enriched in that cluster based on data from Harbison *et al.* (2004) and Macisaac *et al.* (2006). The column Enriched TF Motifs indicates transcription factor consensus motifs found to be enriched in gene promoters of each cluster based on the work of Zhu *et al.* (2009). In both columns, unmarked transcription factors had enrichment with a *P* value less than 0.05, whereas * indicates enrichment with a *P* value less than 0.2, and ** a *P* value greater than 0.2 but less than 0.5. Sliding the *P* value cut off allowed us to include targets that could have been masked because of how they were identified originally; for example, by cut off or condition. The column Enriched GO Terms shows enriched gene ontology terms via MIPS functional or GO biological process categories (see *Materials and Methods*). Those shown were limited to the top six categories (or less if fewer were identified) all with a *P* value at least less than 0.0025. Overrepresented Motifs shows DNA sequences identified in each cluster that were overrepresented in the promoters of those genes. (see *Materials and Methods* for analysis method).

We also looked for short over-enriched motifs within promoter regions of each gene in each cluster using RSAT, an online motif discovery tool for *de novo* promoter enrichment analysis (Thomas-Chollier *et al.* 2008; van Helden 2003). In a related search, we examined transcription factor target enrichments and binding sequence enrichments based on previous genome-wide studies (Macisaac *et al.* 2006; Robert *et al.* 2004; Zhu *et al.* 2009). These enrichments are summarized in Table 1. We find the set of 501 genes induced in common by G, N, and P to be significantly enriched for RRPE (AAAWTTTT) and PAC (GATGAG) elements (Dequard-Chablat *et al.* 1991; Hughes *et al.* 2000; Slattery and Heideman 2007). Of the 501 G-, N-, and P-induced genes, 64% (*P* < 2.3e^−85^) contain at least one RRPE, 53% (*P* < 6.17e^−44^) at least one PAC, and approximately 42% (*P* < 3.67e^−90^) contain both an RRPE and a PAC. This is not surprising in that a connection between genes induced by G repletion and the presence of PAC and RRPE elements has previously been made (Fingerman *et al.* 2003; Hughes *et al.* 2000; Slattery and Heideman 2007; Wade *et al.* 2001).

### Nutrient-specific differences: genes not upregulated in common

In addition to genes displaying a common response, we anticipated nutrient-specific expression patterns. A set of 780 genes met our criteria for G induction, but not for N or P. These transcripts induced 2-fold specifically by G repletion showed a connection to growth metabolism, perhaps distinct from the N and P sets because of the dual energy and carbon source potential provided by G. This set was significantly enriched for RP genes (*P* < 1.42e^−14^), containing 97 of the 137 RP genes (*P* < 3.83e^−52^). As mentioned above, the RP genes were also significantly induced by N or P repletion, but they failed to pass the 2-fold cut off. On closer examination, we found that the RP transcripts had the greater fold induction in G largely because of a greater repression during G starvation (See *Discussion* and Figure S1). In this G-induced set, we found enrichment for gene targets for Rap1, Fhl1, and Sfp1, regulators of RP transcription, and we also found enrichment for their corresponding binding motifs (Fingerman *et al.* 2003; Lieb *et al.* 2001; Marion *et al.* 2004; Pina *et al.* 2003; Schawalder *et al.* 2004). GO enrichments were also observed for translation-related, protein-trafficking, and ER-localization functions (Table 1).

As might be expected, the subset of 115 N-induced genes was enriched for genes involved with amino acid biosynthesis and nitrogen metabolism. This set was enriched for targets bound by Gcn4, Bas1, Met32, Met4, and Cbf1, and it was also enriched for their binding sequence motifs (Table 1).

The 183 genes specifically induced by P were less obviously connected with P metabolism by GO enrichment analysis, appearing enriched for transcriptional control and DNA binding (Table 1). Ino4 and Ino2 targets are enriched, suggesting a need for phospholipid synthesis after P deprivation, an observation noted previously (Greenberg and Lopes 1996).

### Stress genes are downregulated by all three nutrients

**Figure 4:**
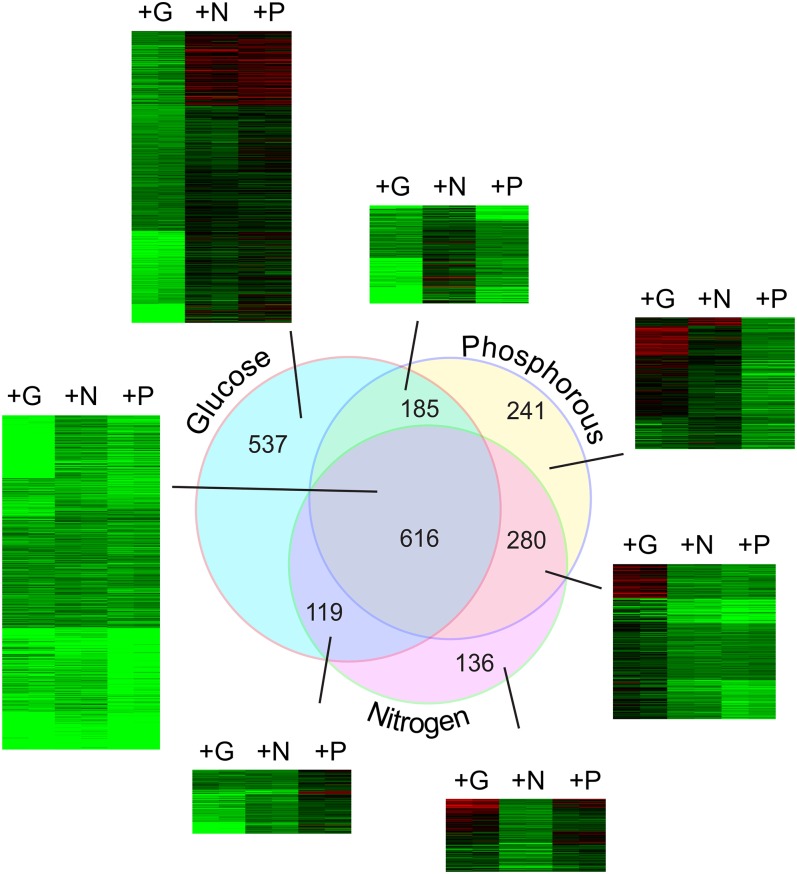
Genes repressed by G, N, and P repletion. Microarray data from Figure 2 was used to identify transcripts reduced by at least 2-fold by G, N or P repletion compared with quiescence levels as described in *Materials and Methods*. The Venn diagram shows the intersections between the sets of genes repressed at least 2-fold by each nutrient. This intersection of three sets produced seven different groups, and a heat map of fold-change responses is shown for each of these seven sets. Independent biological replicate samples are shown as side-by-side columns, and the transcripts are arranged by k-means clustering. The number of individual transcripts in each set is shown in the figure, and the transcripts are listed for each set in Table S2.

We used the cutoff screen described above to identify a set of 616 genes downregulated in common by G, N, or P (Figure 4). Each nutrient decreased the expression of a large group of stress-related genes. Functional classifications showed significant enrichment in the following groups: oxidative stress response (*P* < 6.32e^−9^), heat shock response (*P* < 3.16e^−7^), autoproteolytic processing (*P* < 1.07e^−06^), metabolism of energy reserves (*P* < 3.1^e−5^), and autophagy (*P* < 1e^−14^) (Table 2). We also found this set enriched for peroxisome (*P* < 6.65e^−9^) and vacuolar lumen concentration (*P* < 9.5e^−4^). Perhaps most noteworthy, this set also contains 197 of the approximately 272 (*P* < 1.07e^−134^) environmental stress response (ESR) genes originally identified by Gasch *et al.* (2000). Consistent with this, the group is enriched for STRE elements (Martinez-Pastor *et al.* 1996) in the 5′ intragenic regions (Table 2). This group is also enriched for Hsf1, Msn2, and Msn4 targets important for stress gene regulation (Gasch *et al.* 2000).

**Table 2:** Transcription factor target and binding motif enrichment, gene ontology 25 enrichment, and overrepresented promoter motifs found in the repressed Venn diagram genes

Repressed Cluster	TF Bound	Enriched TF Motifs	Enriched GO Terms	Overrepresented Motifs
G,N,P	HSF1, SN4, MSN2, ROX1, MIG1, STB5, NRG1, SNT2, PHO2^*^, GLN3^*^	MSN2, MSN4, ADR1, SUT1, STP1, GZF3, SKN7, PUT3, MET31, MATA1	Oxidative stress response (32.01.01),	aaccccttaa, ttaagggagc
			Heat shock response (32.01.05),	ttaagggata, cagccgcccttaa
			Autoproteolytic processing (14.07.11.01),	cttcccttaa, agaaggggtt
			Regulation of glycolysis and gluconeogenesis (02.01.03),	ggagta, ccccgg
			Protein/peptide degradation (14.13),	
			Metabolism of energy reserves (*e.g.* Glycogen, trehalose) (02.19)	
G only	GAL4, RCS1, HAP1, YAP1, BAS1, SOK2, SUT1, SKO1, HAP4, HAP3	MIG1, SUT1, ARG80, AFT2, CBF1, RCS1, RTG3, CAD1, PHO4, ADR1	Transcriptional control (11.02.03.04),	accccac
			Sugar transport (20.01.03.01),	ggcggag
			Homeostasis of metal ions (34.01.01.01)	acaaact
			Aerobic respiration (02.13.03),	
			Modification by acetylation, deacetylation (14.07.04),	
			Transcription initiation (11.02.03.01.01),	
N only	STP1, GLN3, DAL82, STB4, GAT1, UME6, DIG1^*^, PUT3^*^, PDR1^*^, YDR520C^*^	UME6, XBP1, GZF3, SOK2, CST6, RTG3, STB5^*^, CRZ1^*^, GAT1^*^, GTS1^*^	Catabolism of nitrogenous compounds 01.02.02.09),	cttatc
			Meiosis I (10.03.02.01),	
			Amine/polyamine transport (20.01.11),	
			Meiotic recombination (10.01.05.03.01),	
			Amino acid/amino acid derivatives transport (20.01.07),	
			Cytoskeleton-dependent transport (20.09.14)	
P only	GCR2, GCR1, TYE7, HAP4, SUM1, RLR1, RTG3, STB2, CBF1^*^, MSN2^*^	GCR1, RCS1, MET4, GCR2, PHO4, ARG81, RAP1, MATA1, BAS1, DIG1	Glycolysis and gluconeogenesis (02.01),	acaaact
			Sugar, glucoside, polyol and carboxylate catabolism (01.05.02.07),	acgtgg
			Cell growth / morphogenesis (40.01),	aggaag
			C-compound and carbohydrate metabolism (01.05),	
			Purine nucleotide/nucleoside/nucleobase anabolism (01.03.01.03)	
G, N	THI2, CIN5, STB1^*^, UME6^*^, YAP7^*^, GAT1^**^, ARR1^**^, CST6^**^, ARG80^**^, PHD1^**^	SUT1, GTS1, DIG1, SOK2, STE12, GAT1, GZF3, IME1, MSN2, MET31	Development of asco- basidio- or zygospore (43.01.03.09),	gcccct
			Meiosis (10.03.02),	tccgcagg
			Amino acid/amino acid derivatives transport (20.01.07),	
			Allantoin and allantoate transport (20.01.23),	
			Autophagy (GO:0006914)	
G, P	MET31,MSN4 MET4, MSN2 GCN4, ACE2, BAS1 MET32, CBF1, UGA3	MSN2, MSN4, MIG1, BAS1, ARG81, XBP1, CRZ1, GZF3, STB4, UGA3	Sugar, glucoside, polyol and carboxylate catabolism (01.05.02.07),	gcaggggt
			Tricarboxylic-acid pathway (02.10),	cggagc
			Electron transport and membrane-associated energy conservation (02.11),	cccgcc
			Stress response (32.01),	cagccg
			Aerobic respiration (02.13.03)	
N, P	RPN4, FKH2, FKH1,NDD1, MSN2,MSN4, SUT1^*^, CIN5^*^, REB1^*^, HSF1^*^	RPN4, YHP1, MSN2, SUT1, GAT3, HSF1, NDD1, IME1^*^, MSN4^*^, ADR1^*^	Proteasomal degradation(14.13.01.01),	aaggga
			Modification by ubiquitination, deubiquitination (14.07.05),	
			Vacuolar/lysosomal transport (20.09.13),	
			Actin cytoskeleton (42.04.03),	
			ATP binding (16.19.03),	
			Tetracyclic and pentacyclic triterpenes (cholesterin, steroids and hopanoids) metabolism (01.06.06.11),	

Each of seven gene clusters identified in Figure 3B was searched for transcription factor target gene enrichment, transcription factor motifs in gene promoter regions, gene ontology (GO) enrichment, and overrepresented promoter motifs. Otherwise, columns are as described in Table 1.

### Nutrient-specific differences: nutrient-specific downregulation

We found 537 genes downregulated by G repletion but not N or P. This group was enriched for sugar transport (*P* < 3.15e^−8^), aerobic respiration (*P* < 3.5e^−4^), transcriptional control (*P* < 1.92e^−8^), and homeostasis of metal ions (*P* < 2.6e^−4^). This cluster was enriched for gene products localizing to the mitochondrial inner membrane, consistent with GO enrichment for aerobic respiration. Gal4, Hap1, Sok2, and Sut1 targets were also enriched in this set, and motifs for Mig1 and Rtg3 were enriched in the 5′ intragenic regions (Table 2).

The 136 genes specifically downregulated by N alone were enriched for catabolism of nitrogenous compounds (*P* < 8.29e^−5^) and amino acid/amino acid derivative transport. While this set was enriched for nitrogen catabolite repression (NCR) genes (*P* < 1.9e^−9^), the majority of NCR genes were found in the set repressed in common by G, N, and P, an observation in concordance with the fact that many NCR genes are elevated in starvation (Wu *et al.* 2004). Finally, the 5′ intragenic regions of this cluster were enriched for Stp1, Gln3, Bas1, Gat1 binding targets, and their corresponding sequence motifs.

Interestingly, the set specifically repressed by P was enriched for genes associated with glycolysis and gluconeogenesis (*P* < 7.084e^−11^), as well as sugar, glucoside, polyol, and carboxylate catabolism (*P* < 9.988e^−7^). The 5′ intragenic regions of this set were enriched with Gcr1, Gcr2, and Hap4 targets. As expected, the set was also enriched for Pho4 targets; however, Pho4 target genes were spread between several repressed clusters, especially the common G, N, P cluster.

### Requirement for cAMP and TOR

The large transcriptional response to G is dependent on sensory pathways requiring Gpa2, cAMP/PKA, and to a lesser extent, TOR signaling (Slattery *et al.* 2008; Wang *et al.* 2004). Addition of G to post-log cells has long been linked to activation of PKA via cAMP (Matsumoto *et al.* 1985). However, the receptors that link G signals to cAMP production are quite distinct from those that sense N and P (Zaman *et al.* 2008). Nonetheless, the similarities between the transcriptional responses to G, N, and P suggested that PKA and TOR might be involved in the responses to all three nutrients.

**Figure 5:**
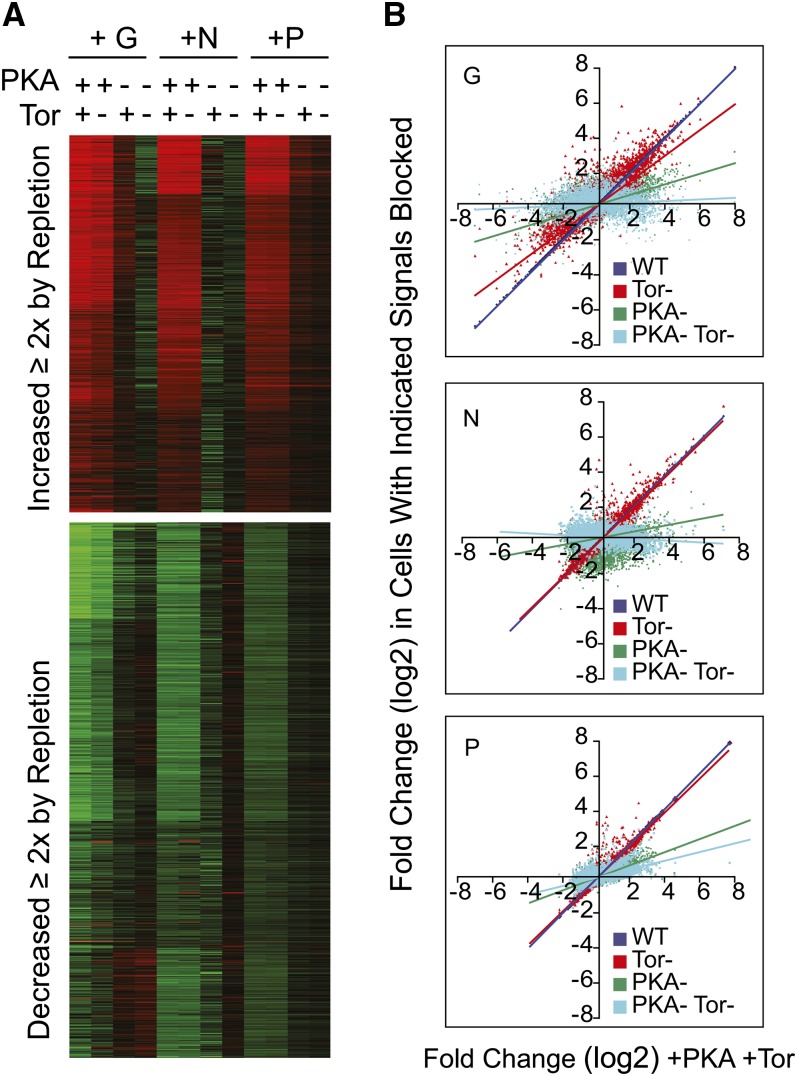
The effect of PKA and TOR signaling in the transcriptional response to G, N, and P. Cells carrying a *cyr1*Δ mutation (TC41) were starved for G, N, or P until growth arrested, and cAMP was removed as described in *Materials and Methods*. Samples for microarray analysis were taken at the starved state and 60 min after repletion of each missing nutrient. In some samples, rapamycin (200 nM) was added during repletion to block the TOR pathway, and in some samples, cAMP (1 mM) was omitted from the repletion medium to block the cAMP/PKA pathway. (A) Heat map showing the average log2 fold change for each of the 501 (top panel) or 616 genes (bottom panel) identified in Figures 3 and 4 as induced or repressed in all three nutrient-repletion conditions. Heat maps were k-means clustered. Red indicates induction relative to the starved state; green indicates repression; and black, no change. The + symbols indicate active and the − symbols indicate blocked pathways, produced by adding and subtracting rapamycin and cAMP. (B) For each transcript, the average fold change in expression induced by nutrient repletion in the *cyr1*Δ mutant +cAMP and −rapamycin is plotted on the X-axis (+PKA, +TOR). Responses under different conditions are shown on the Y-axis as follows: red is fold change produced in *cyr1*Δ cells treated with rapamycin (−TOR); green is fold change produced in *cyr1*Δ cells without cAMP (−PKA); light blue is fold change produced by nutrients in *cyr1*Δ cells without cAMP and with rapamycin (−PKA-TOR); and dark blue is fold change produced by nutrients in the isogenic wild-type cells (HR125). The slope of each line indicates the severity of signaling inhibition across the genome relative to the +PKA, +TOR samples.

To test this, we selectively blocked PKA, TOR, or both, and we measured the effect on the transcriptional response to repletion of G, N, or P. We blocked cAMP production using a *cyr1Δ* strain (TC41) and blocked TOR with rapamycin (Mitts *et al.* 1990; Russell *et al.* 1993; Slattery *et al.* 2008). For each of the three nutrients, both gene induction and repression were highly dependent on cAMP/PKA and TOR. The heat map in Figure 5A focuses on the core 501 upregulated and 616 downregulated genes responding to G, N, or P. Loss of both PKA and TOR signaling essentially blocked the large-scale transcriptional response to nutrient repletion. We found that most transcript changes showed a greater dependence on cAMP/PKA than on Tor.

The plots in Figure 5B compare the responses to nutrient repletion between cells with normal cAMP/PKA and TOR with the responses in cells when different pathways are blocked. Each transcript on the array is plotted as a dot, with the X-axis position indicating the fold change caused by nutrient repletion with both TOR and cAMP/PKA intact; the position on the Y-axis indicates the response when the indicated pathways are blocked. The different pathway manipulations are represented by different colors, and each nutrient repletion, G, N, or P, is presented as a separate graph.

As a control, we plotted the response to each nutrient in a wild-type *CYR1*^+^ strain against our *cyr1*Δ strain supplied with cAMP. This produced an almost perfect diagonal line, shown as dark blue; the line obscures the dots. This indicates that our *cyr1*Δ mutant can respond normally when cAMP is provided.

TOR blockade (red dots) caused some loss of response, while loss of cAMP (dark green) had a striking impact on the response to each nutrient. With both TOR and PKA knocked out (turquoise), the responses to nutrient addition were substantially reduced: the G and N responses were almost completely blocked, and a small amount of the response to P repletion remained.

### Nutrient signaling through cAMP

Although the connection between glucose and cAMP production is well known (Boy-Marcotte *et al.* 1987; Camonis *et al.* 1986; Matsumoto *et al.* 1982; 1985; Thevelein and Beullens 1985), we were surprised to find that the response to N and P repletion was cAMP-dependent. If N and P produce the transcriptional response through cAMP/PKA, then directly stimulating this pathway by adding cAMP should bypass the nutrients and replicate the response. On the other hand, cAMP might be necessary without playing a direct role in transmitting the signal. In this permissive role, cAMP alone should be inactive without N or P.

**Figure 6:**
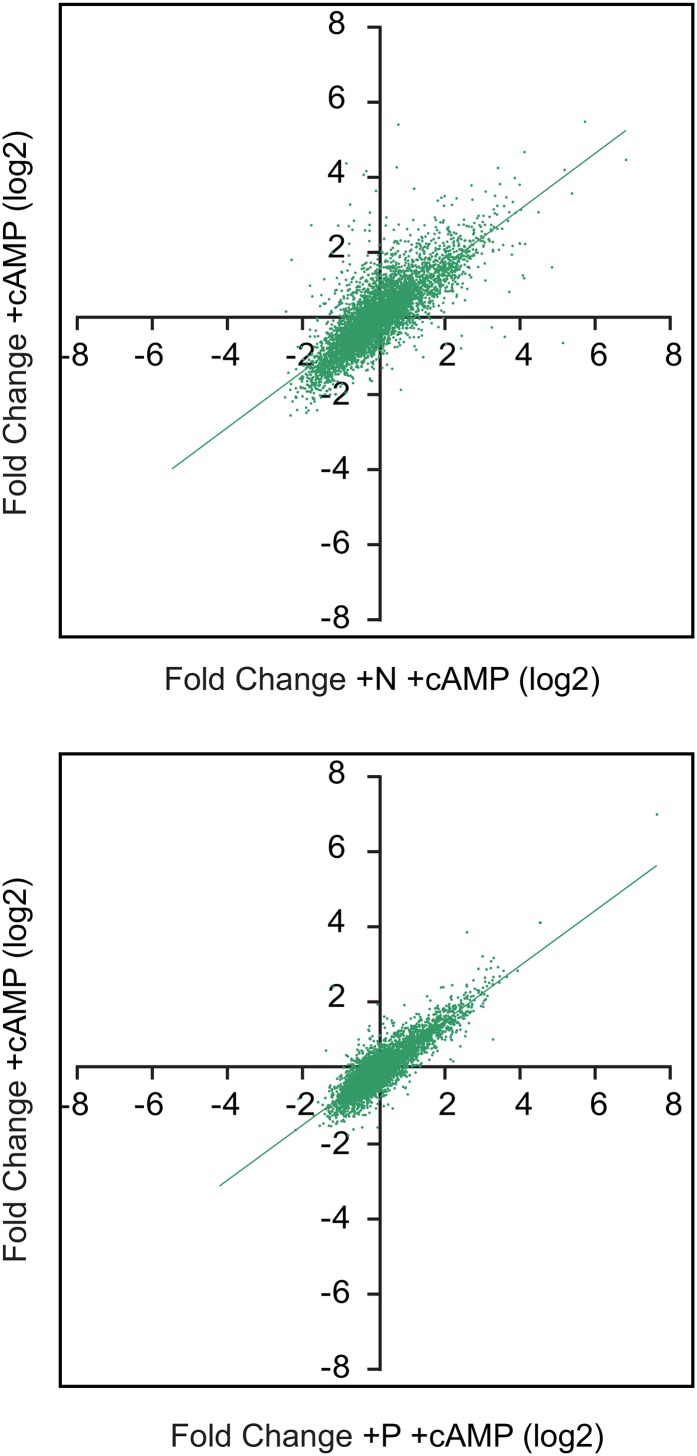
Response of N- or P-starved cells to cAMP addition. Cells carrying a CYR1 deletion (TC41) were grown until limited for N or P as described in *Materials and Methods*, and then challenged with either cAMP alone or cAMP with the limiting nutrient. Samples were collected at the starved state and 60 min after repletion for microarray analysis. For each transcript, the average fold change in expression induced by cAMP plus nutrient is plotted on the X-axis, and the response to cAMP alone is plotted on the Y-axis. Comparison of the response to cAMP alone with cAMP plus N produced a Pearson correlation of 0.79, and comparison of cAMP alone with cAMP plus P yielded a correlation of 0.87.

To test this, *cyr1*Δ cells were N or P depleted with cAMP present to ensure growth into quiescence, and then moved to starvation minus cAMP for 24 hr. The cultures were then divided, and the missing nutrient plus cAMP was added to one aliquot, while cAMP alone was added to the other, and then the samples were prepared for microarray analysis. Changes in gene expression produced by cAMP alone are plotted on the Y-axes, and the responses to the nutrient with cAMP on the X-axes (Figure 6). The response to cAMP produced transcriptional changes that were largely similar to those produced by the nutrient itself with cAMP present. The correlation between cAMP alone and cAMP and N was 0.79, while the correlation with cAMP and P was 0.87.

Of note is the fact that when cAMP alone was added, these cells did not grow and under the microscope maintained a quiescent appearance. The transcriptional response is not sufficient to produce growth in the absence of the missing nutrient.

Thus, the responses to N and P were largely cAMP-dependent. Furthermore, cAMP was able to produce similar transcriptional responses to those produced by N or P repletion. We have previously observed very similar results with G repletion (Slattery *et al.* 2008).

**Figure 7:**
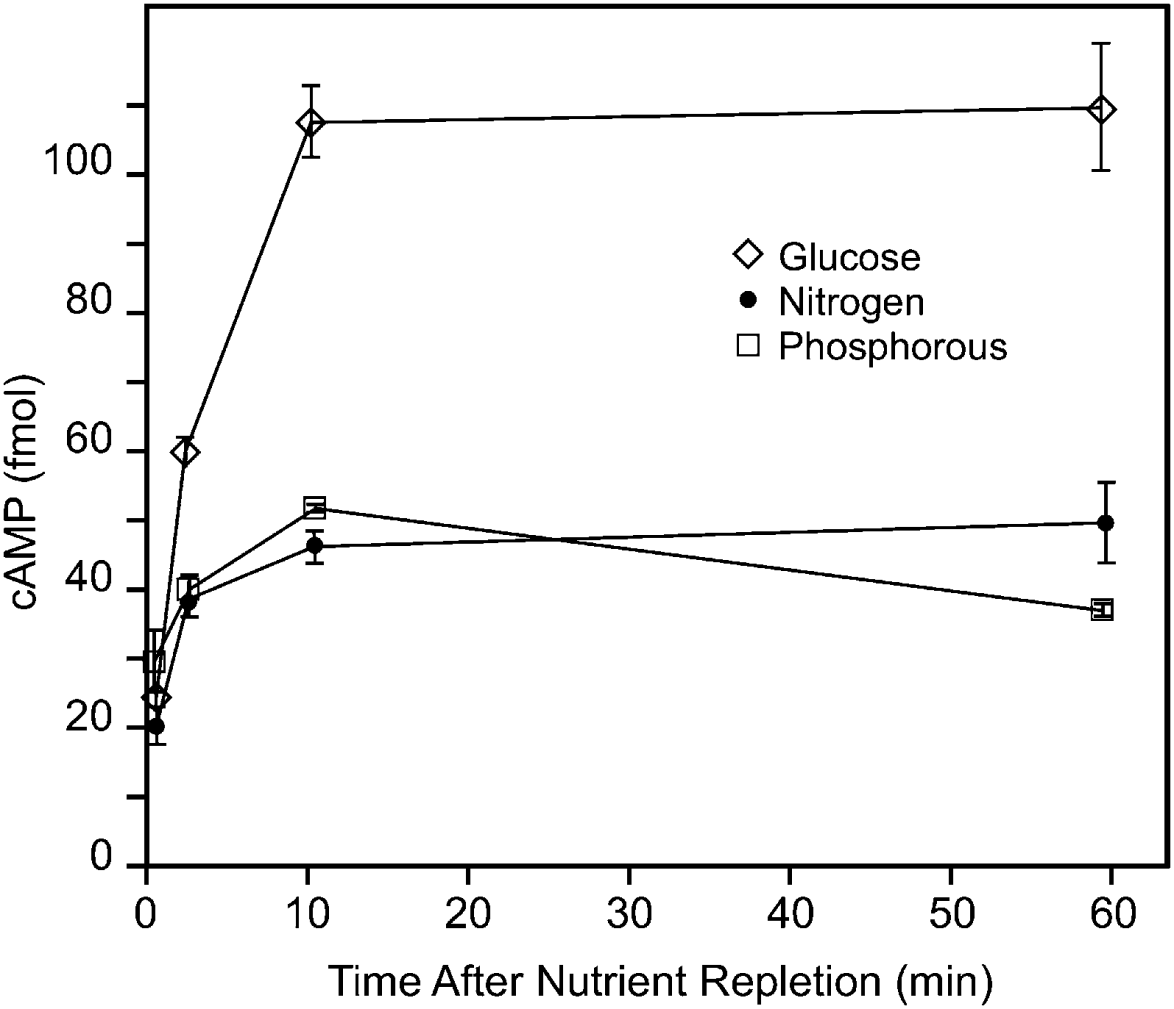
G, N, and P repletions increase cAMP. Prototrophic yeast (S288C) were starved for G, N, or P and then repleted with the missing nutrient as described in *Materials and Methods*. Levels of cAMP were measured at the indicated time after repletion using an ELISA assay, with each well receiving extract from the same number of cells (5 OD units). Error bars represent the standard error the mean (n = 3). The experiment was repeated multiple times with similar results.

The obvious next step was to examine the effect of G, N, and P repletion on cAMP levels. While glucose tended to produce a more robust response, in repeated experiments, we consistently found that all three nutrients increased cAMP levels when added back to quiescent cells (Figure 7). The sustained cAMP levels following G repletion are consistent with our previous results (Russell *et al.* 1993).

### Cross talk between nutrient signals

The fact that cAMP is required for the transcriptional responses to G, N, and P, that the nutrients produce increases in cAMP, and that cAMP alone can simulate the repletion response indicates that nutrients regulate these large changes in part through cAMP/PKA. However, this model poses problems: How can a cell halt growth when just one nutrient is depleted? How does the cell know which nutrient is sending the signal? Indeed, we wondered whether there were cases where adding one nutrient could substitute for the lack of another.

**Figure 8:**
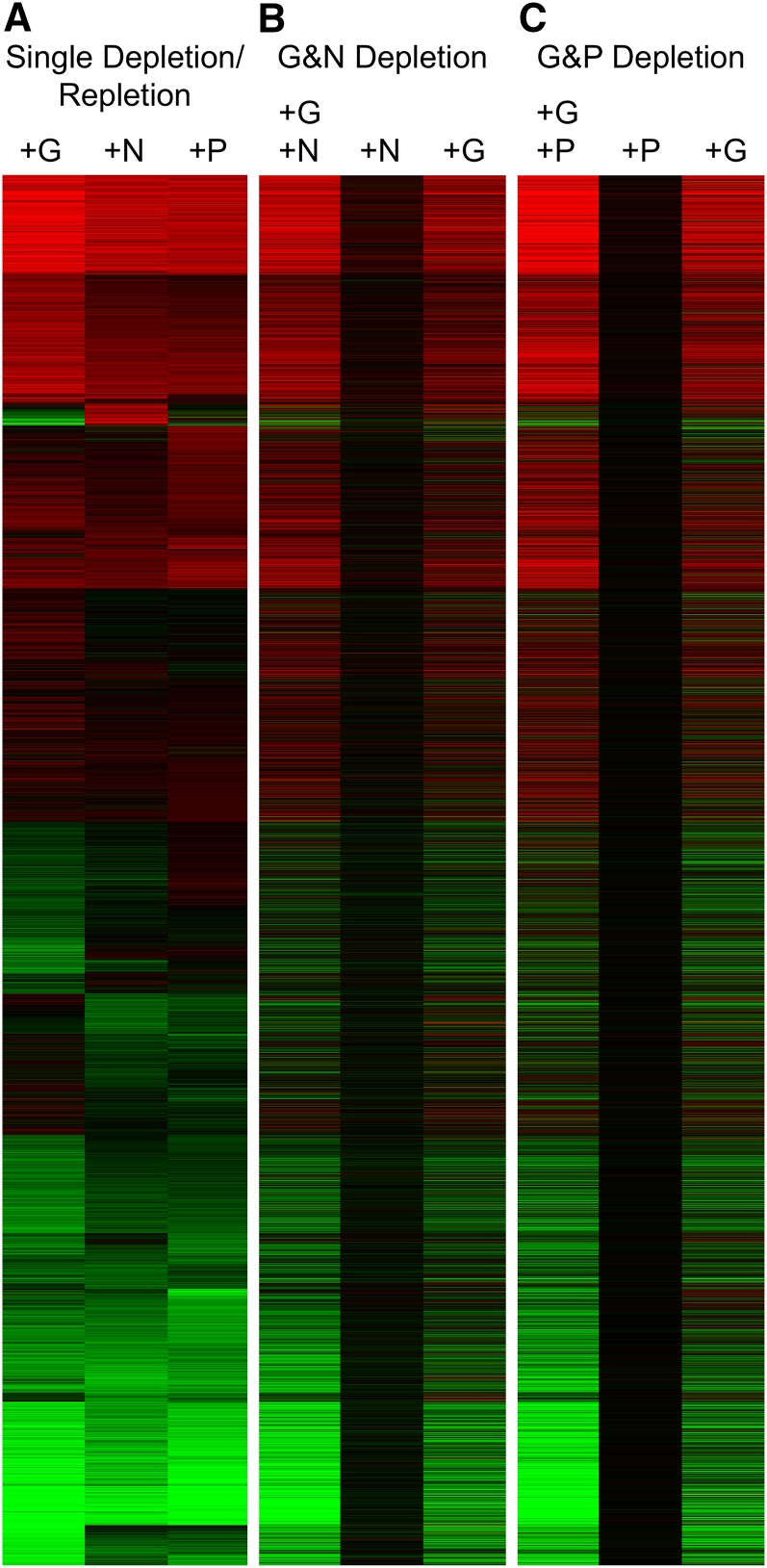
Cross talk between nutrient signals. S288C cells were starved for the indicated nutrients and repleted with either a single nutrient or both of the limiting nutrients as described in *Materials and Methods*. (A) As a point of reference, the results for depletion and repletion of single nutrients (G, N, and P) are shown, and the k-means clustering pattern of this set of single nutrient repletions was used to order the other two panels. (B) Cells limited for both G and N were repleted with G, N, or both, and then cells were collected for microarray analysis 60 min after nutrient addition. Duplicate experiments were conducted, and the heat map shows the average log2 fold-change ratio induced by the nutrients for each transcript. Red indicates induction relative to the starved state; green indicates repression; and black, no change. (C) Samples were depleted for both G and P and repleted as indicated.

To examine this, we simultaneously depleted cells for two nutrients, and then repleted only one of the pair. We measured changes in transcripts when we added each nutrient alone or with its missing partner. For comparison, we also repeated the depletion experiments in which a single nutrient was depleted and repleted (Figure 8A).

As expected, The GP- and GN-depleted cells showed robust responses to repletion of both limiting nutrients together (Figure 8, B and C). These responses were largely similar to those studied earlier when a single nutrient was depleted and repleted (Figure 8A). However, addition of N alone to a GN-depleted culture produced almost no response; nor did addition of P to a GP-depleted culture. In contrast, addition of G alone, while not producing growth, produced a large transcriptional response in the doubly depleted cultures, despite the fact that the cells were still growth limited by N or P.

### Origin of nutrient signals

**Figure 9:**
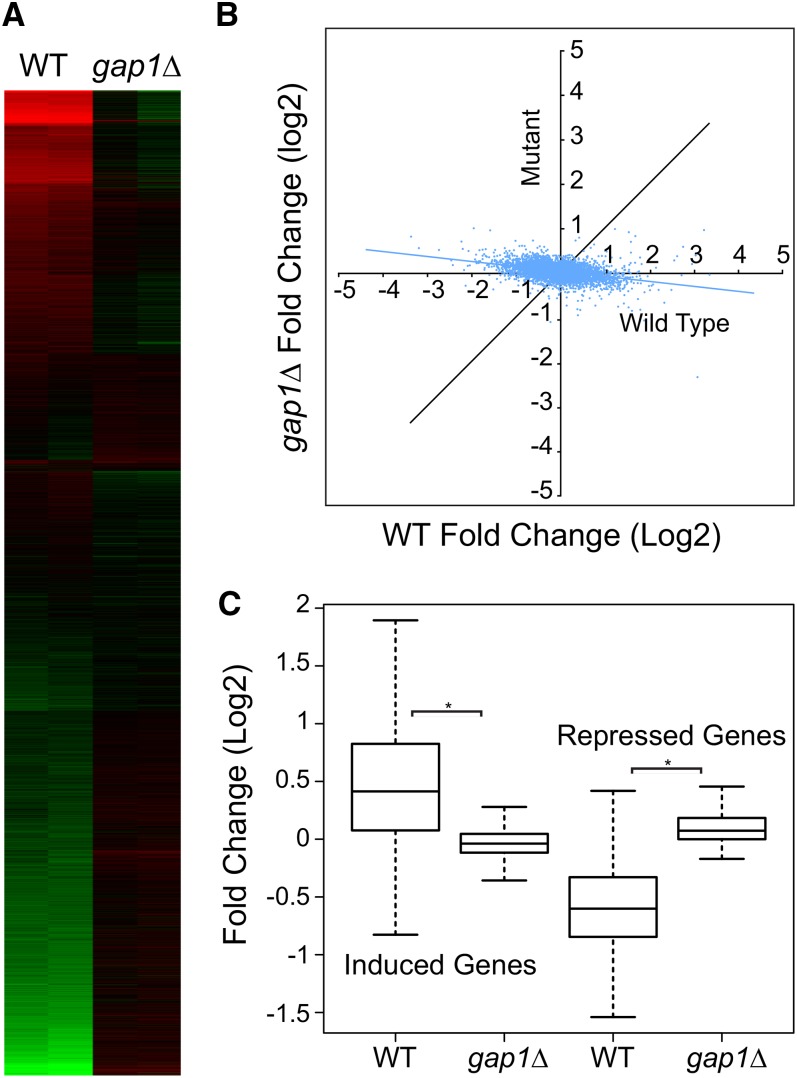
Effect of *GAP1* deletion on the transcriptional response to N. Wild-type (BY4742) or the isogenic *gap1Δ* cells were starved for N as in Figure 1 and then challenged with 10 mM L-citrulline. Samples for microarray analysis were collected at the starved state and 60 min after L-citrulline repletion. (A) The heat map shows the log2 fold-change results for each transcript of duplicate independent experiments in parallel columns: red indicates induction relative to the starved state, and green indicates repression. The k-means method was used to arrange gene clusters. (B) Dot plot in which the average log2 response to L-citrulline is plotted for each transcript. The fold-change response in wild-type cells is plotted on the X-axis, and the response in the *gap1*Δ mutant is shown on the Y-axis. (C) Box plots summarizing the expression of the 501 induced or 616 repressed genes from Figures 3 and 4 in either the wild-type or the *gap1*Δ strains. The black band in the box indicates the median; the upper box limit, the 75^th^ percentile; the lower box limit, the 25^th^ percentile; and each whisker, the minimum and maximum value within 1.5× of the interquartile range, respectively. The asterisk indicates a significant difference (*P* < 0.05).

Gap1 activates trehalase in response to amino acid stimulation (Donaton *et al.* 2003). To determine whether Gap1 carries signals regulating the large-scale response to nitrogen repletion, we challenged wild-type and *gap1*Δ cells with citrulline and then collected RNA for microarray analysis. We used citrulline because it is a relatively specific Gap1 agonist (Donaton *et al.* 2003; Grenson *et al.* 1970). We found that addition of citrulline to N-depleted wild-type cells produced a marked change in transcript levels relative to the starved state as shown in the heat map in Figure 9. However, this response was almost entirely silenced by loss of *GAP1*, indicating that the signal induced by citrulline repletion requires Gap1.

The dot plot in Figure 9B shows for each transcript the fold change produced by L-citrulline plotted on the X-axis for wild-type cells and on the Y-axis for the *gap1*Δ mutant samples. For most transcripts that responded to citrulline, loss of Gap1 had a profound effect.

The box and whisker plots in Figure 9C show the effect of citrulline on the sets of 501 and 616 core genes induced or repressed at least 2-fold by G, N, or P in Figures 3 and 4. It can be seen that the responses produced by L-citrulline average less than 2-fold, so L-citrulline produces a less robust response than the original N repletion, which included both ammonium and amino acids. However, more than 75% of the 501 genes in this set were induced by citrulline, and this was almost completely dependent on Gap1. Repression was also Gap1-dependent.

The ammonium permeases Mep1, Mep2, and Mep3 are all involved in ammonium activation of trehalase, although Mep2 produces the most prominent signal (Van Nuland *et al.* 2006). Mep2 has also been implicated in regulating the ammonium control of pseudohyphal growth (Lorenz and Heitman 1998).

**Figure 10:**
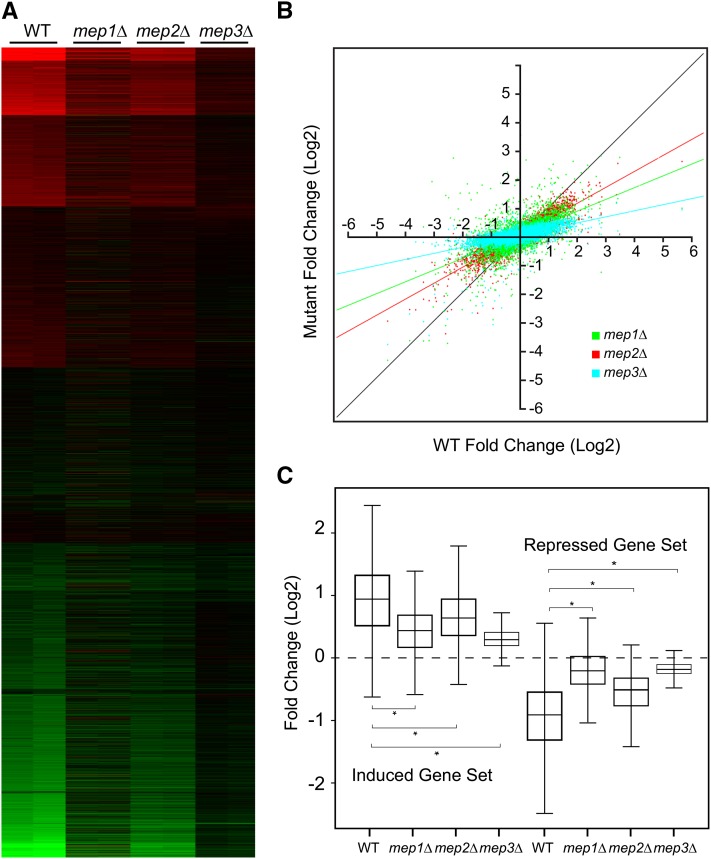
Role of Mep transceptors in the response to N. Wild-type (BY4742) and the isogenic *mep1Δ*, *mep2Δ*, and *mep3Δ* cells were starved for N, and then challenged with 10 mM ammonium sulfate. Samples for microarray analysis were taken at the starved state and 60 min after repletion. (A) The heat map shows the results of duplicate experiments presented in parallel columns for the wild-type and the *MEP* mutants. Results are shown as log2 fold change, with red indicating induction by ammonium, and green repression relative to the initial quiescent samples. The patterns were obtained using k-means clustering. (B) Dot plot with log2 fold change produced by ammonium in wild-type plotted on the X-axis, and the responses in each of the *MEP* mutants plotted on the Y-axis. Each dot represents a gene, and the black line indicates the pattern expected for a perfect correlation. The *mep1*Δ response is shown in green; the *mep2*Δ , in red; and the *mep3*Δ, in light blue. (C) Box plots summarizing the results for the core sets of 501 induced and 616 repressed genes as in Figure 9C. The black band in the box indicates the median; the upper box limit, the 75^th^ percentile; the lower box limit, the 25^th^ percentile; and each whisker, the minimum and maximum value within 1.5× of the interquartile range, respectively. The asterisk indicates a significant difference (*P* < 0.05).

As in the previous experiment, we used N-starved wild-type or *mep1Δ*, *mep2Δ*, or *mep3Δ*, mutant cells and challenged them with 10 mM ammonium. The heat map shows that each mutation has some impact on the overall response to ammonium, with the *mep3Δ* mutant producing the most blunted response (Figure 10). The relative impacts that the mutations had are shown in the dot plot in Figure 10B, and the effects on the 501 induced and 616 repressed genes described above are shown in the box and whisker plots of Figure 10C. Overall, it appears that, as with trehalase, all three *MEP* genes contribute to the transcriptional response to ammonium repletion.

**Figure 11:**
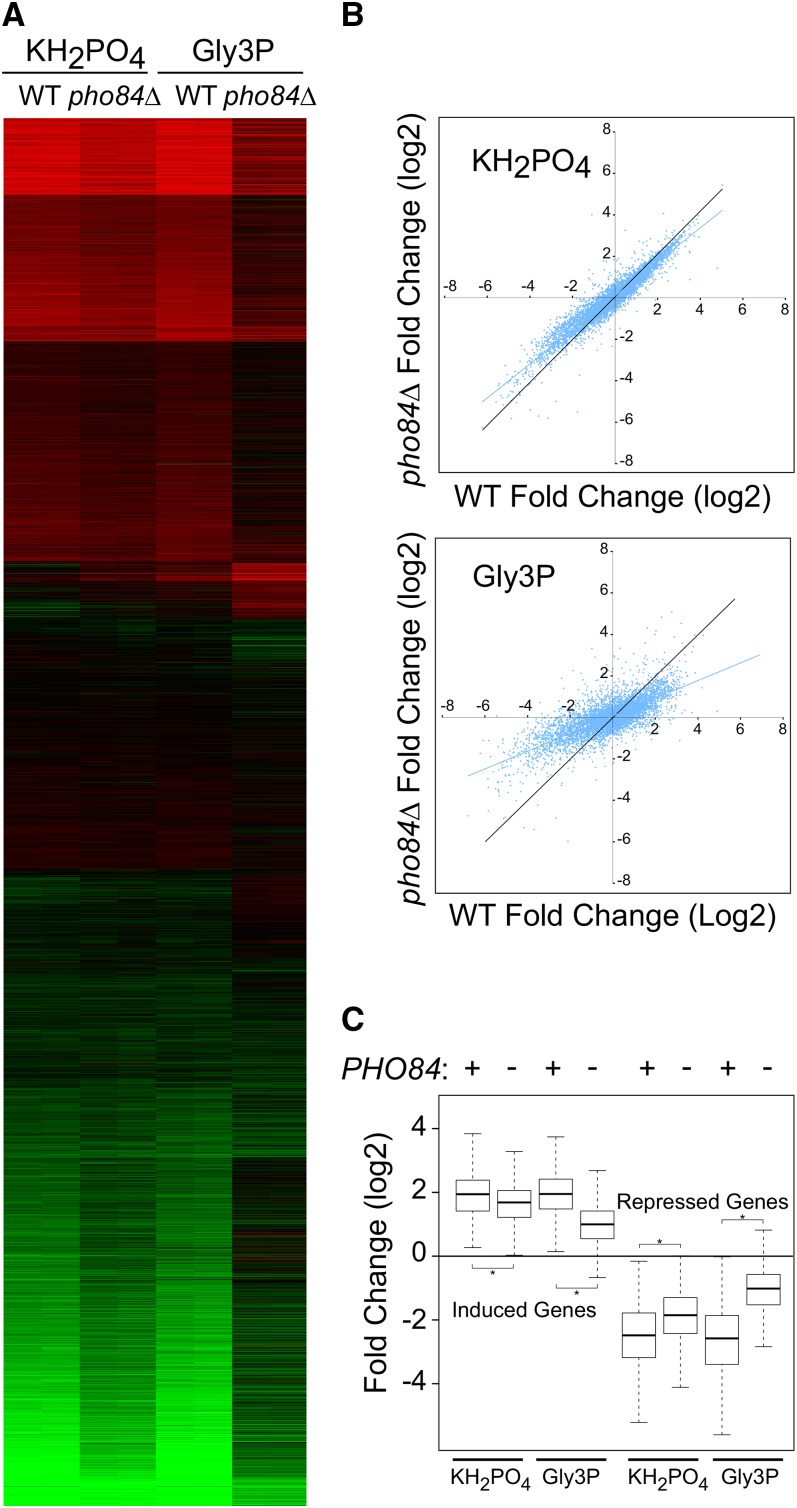
Role of *PHO84* in the transcriptional response to P. Wild-type (BY4742) or isogenic *pho84Δ* cells were starved for P, and then challenged with KH_2_PO_4_ or Gly3P as described in *Materials and Methods*. Samples for microarray analysis were taken from the initial quiescent culture and at 60 min after repletion. (A) The heat map shows the results of duplicate experiments presented in parallel columns for the wild-type and the *pho84Δ* mutants. Results are shown as log2 fold change, with red indicating induction by the P source, and green repression relative to the initial quiescent samples. The patterns were obtained using k-means clustering. (B) Dot plots showing responses to KH_2_PO_4_ and Gly3P in wild-type and pho84Δ mutant cells. The log2 fold change for the wild-type cells is plotted on the X-axis, and the response in the *pho84Δ* mutant is plotted on the Y-axis. Each dot represents a gene, and the black line indicates the pattern expected for a perfect correlation. (C) Box plots summarizing the results for the core sets of 501 induced and 616 repressed genes as in Figures 9C and 10C. The presence of the wild-type *PHO84* gene is indicated by a + sign. The black band in the box indicates the median; the upper box limit, the 75^th^ percentile; the lower box limit, the 25^th^ percentile; and each whisker, the minimum and maximum value within 1.5× of the interquartile range, respectively. The asterisk indicates a significant difference (*P* < 0.05).

The Pho84 phosphate transporter has been reported to act as a phosphorous sensor (Popova *et al.* 2010). We challenged wild-type and *pho84*Δ cells with two P sources: KH_2_PO_4_, the a Pho84-transportedP source used in the previous repletions, and glycerol-3-phosphate (Gly3P) as a specific agonist for Pho84 (Popova *et al.* 2010). We found that loss of *PHO84* produced different effects depending on the P source. As shown by the heat maps and dot plots in Figure 11, the response to Gly3P was considerably attenuated by loss of *PHO84*, while the KH_2_PO_4_ response was not.

Figure 11C shows a box and whisker plot focused on the sets of 501 and 616 transcripts upregulated and downregulated, respectively, by all three nutrients. The effect of *PHO84* deletion on the KH_2_PO_4_ response was quite subtle: the response to Gly3P repletion was more distinct. Overall, these data suggest that *PHO84* plays a role in, but is not solely responsible for, signaling the transcriptional response to P.

## Discussion

### Growth and quiescence

The ability of yeast cells to cease division and attain a stress-resistant state in response to different nutrient limitations was recognized some time ago (Lillie and Pringle 1980; Paris and Pringle 1983). This somewhat commonplace occurrence has several implications. First, it appears that quiescence and growth, while influenced by different conditions, are two fundamental states for yeast cells. This conclusion was recently strengthened by Klosinska *et al.* (2011). Another implication is that cells must use different nutrient signals to control entry into growth and quiescence.

A simple idea would be that as any nutrient becomes limiting, metabolic activity subsides and that drop is sensed. While this plan relieves the cell of having to sense many different important nutrients, two arguments can be made against it. First, allowing critical resources to reach such low levels so as to limit metabolism would be expected to substantially reduce fitness; in fact, nutrients are stored prior to quiescence. Perhaps more compelling are the numerous nutrient-sensing mechanisms that are now being discovered. Indeed, many of the responses described in this article can be observed under conditions in which the cell cannot, or does not, metabolize the nutrient producing the signal.

One important difference that we noted between nutrient-limited states was that cells deprived of glucose respond more conservatively than those limited for N or P. This is best observed in the group of 137 RP genes, which are considerably more repressed when cells are limited for G than by N or P. In fact, it was because of this failure to repress the RP genes during N or P starvation that the N induction of this set by N or P failed to make the ≥ 2× cut off (Figure 3 and Figure S1). A similar effect was observed with the 230 RiBi transcripts, being on average about 2-fold more repressed in G-depleted cells than in N- or P-depleted cells (Figure S1). This effect contributes to the observation that the fold changes produced by N and P resembled each other more closely than they resembled the response to G.

While N and P are needed primarily as elemental building blocks, limitation for G affects both the availability of carbon and cellular energy. We speculate that because cells are adept at recycling nutrients as long as energy is plentiful, they can afford to maintain higher levels of RP and RiBi gene expression if G is present than they do if starved for G and the energy G provides. The RP and RiBi transcripts represent a large fraction of the total mRNA.

### A common response triggered by different chemical signals

Glucose has been studied in relation to growth, metabolic regulation, respiration, and glucose repression (Carlson 1999; Hedbacker and Carlson 2008). Many studies of N signalng have focused on the role of N as a regulator of metabolic processes requiring nitrogen as an element. Many studies have focused on nitrogen metabolism and the TOR pathway (Cardenas *et al.* 1999; Cooper 2002; Crespo *et al.* 2002; Hardwick *et al.* 1999; Hinnebusch 2005). A similar approach has been used in studying P signals, focusing on phosphate in metabolism (Wykoff and O’Shea 2001). This has produced models in which G, N, and P initiate signals in yeast through very distinct pathways (Forsberg and Ljungdahl 2001; Magasanik and Kaiser 2002; Schneper *et al.* 2004). With this in mind, it was possible that the transcriptional responses might converge in a minor way but be largely distinct and non-overlapping.

A set of experiments by Thevelein *et al.* (2005) indicated that all three nutrients were tied together in a PKA-dependent process (Giots *et al.* 2003; Popova *et al.* 2010; Van Zeebroeck *et al.* 2009). However, this work left two points in question. First, trehalase activation, while providing an indirect measurement of PKA activity, does not shed light on the scope of the response. Second, only G, and not N or P, was thought to increase cAMP.

We found that the response to G, N, and P involves a core set of at least 1117 genes: approximately 18% of the yeast genome. We conclude that the need to induce genes for growth and to repress unneeded stress genes is compelling and generalized. The gene set induced was enriched for ribosome biogenesis and translation genes; these would be required for growth regardless of the initiating nutrient signal. By the same token, the set of 616 repressed genes was enriched with stress response genes (Gasch *et al.* 2000). Cells initiating growth in newly repleted medium under favorable conditions are best off putting their energy into other processes and reducing stress-response transcripts, again regardless of the repleted nutrient.

### Role of cAMP and PKA

Glucose induces cAMP production (Boy-Marcotte *et al.* 1987; François *et al.* 1987; Russell *et al.* 1993). However, while N and P have been shown to produce responses indicative of PKA activation, there has been little if any positive link between the repletion of these nutrients and cAMP production (Donaton *et al.* 2003; Popova *et al.* 2010; Van Nuland *et al.* 2006; Van Zeebroeck *et al.* 2009). In our hands, all three repletions consistently produced increases in cAMP levels that were sustained over an hour. This is consistent with the trehalase activation as well as the cAMP requirement for the large-scale transcriptional response to all three nutrients and the ability of cAMP itself to mimic nutrient repletion in the absence of the missing nutrient.

Clearly, induction and repression of mRNAs is not enough to make cells grow: metabolism, protein synthesis, and many other processes are required in parallel. Thus, simply creating a large transcriptional response by itself without also providing the missing nutrient did not produce growth.

While the TOR pathway is clearly important in nutrient signaling (Cardenas *et al.* 1999; Cooper 2002; Wullschleger *et al.* 2006), we found TOR inhibition produced a smaller overall effect than loss of cAMP production. In fact the largest effect was observed when both pathways were blocked, suggesting overlapping and partially redundant roles for the two kinase pathways (Martin *et al.* 2004; Roosen *et al.* 2005; Santangelo 2006; Zurita-Martinez and Cardenas 2005).

### Transceptors

The response to G repletion can be explained by stimulation of the glucose receptor Gpr1, coupled to the Gpa2 G-protein to stimulate cAMP production by adenylyl cyclase, encoded by *CYR1*. While not fully understood, this mechanism of cAMP generation has long been studied (Santangelo 2006; Zaman *et al.* 2008, 2009). The connections between N, P, and cAMP/PKA are less well understood.

Gap1 has recently been discovered to produce signals in response to amino acid substrates to control Gap1 processing as well as trehalase activation (Cain and Kaiser 2011; Kriel *et al.* 2011; Merhi *et al.* 2011; Van Zeebroeck *et al.* 2009). Citrulline has been identified as a specific Gap1 agonist that can generate a signal inducing trehalase without being metabolized as a nitrogen source (Donaton *et al.* 2003). We found the response to L-citrulline was Gap1-dependent. Therefore, in addition to regulating the PKA-dependent posttranslational increase in trehalase, we found that Gap1 plays a role in our cAMP-dependent transcriptome response to N repletion.

Mep2 is involved in controlling pseudohyphal growth and trehalase activation (Rutherford *et al.* 2008; Van Nuland *et al.* 2006). Therefore, Mep protein involvement in the transcriptional response to ammonium is not surprising. We found that each of the Mep1–3 proteins played a role in the response to ammonium.

There has also been growing evidence that Pho84 can serve as a transceptor regulating both aspects of phosphate metabolism and PKA-dependent trehalase activation (Giots *et al.* 2003; Lagerstedt *et al.* 2004; Lundh *et al.* 2009; Thevelein *et al.* 2005). Gly3P has been found to be a transceptor agonist that can activate Pho84 without transport by Pho84 (Popova *et al.* 2010). Loss of Pho84 had little impact on the response to orthophosphate repletion, but it did inhibit the response to Gly3P. Because Gly3P signaling is largely Pho84-dependent, this suggests that the receptors for orthophosphate are redundant: other receptors maintain the response in the absence of Pho84. We speculate that Gly3P is not a good agonist for the redundant receptors.

Prior to the advent of microarrays, a prevalent idea was that yeast growth fueled transcription in general. We show that large changes in mRNA abundance precede growth rather than result from growth and that they are readily decoupled from growth. For example, both citrulline and Gly3P are known agonists for their respective transceptors, yet they are poor nutrients. However, both of these agents produced a large-scale response. A strain unable to take in and grow on glucose still produces a normal large-scale transcriptional response to G repletion (Slattery *et al.* 2008). In dually starved cells, addition of G alone produced a transcriptional response that was not sufficient to induce growth. Finally, the addition of cAMP mimicked the nutrient repletion responses in cells that remained nutrient depleted. These results are evidence for a sensing system that can directly determine when a missing nutrient is restored.

### Cross talk

Because G, N, and P each appear to signal through PKA in a cAMP-dependent manner, it is not clear how the cell can differentiate between the nutrients. In fact, one would predict that cross talk would occur. We found some evidence for cross talk in which G alone induced transcriptional responses in dually GN- or GP-limited cells. N or P alone did not produce a response when G was absent. We have found that G upshifts can produce some transcriptional responses in N- or P-limited cultures (data not shown). Griffioen *et al.* (1996) observed that G upshift produced RP gene induction that was initially independent of growth. With the information available at present, we conclude that the three nutrients examined produce a common response through mechanisms that have common elements, yet in at least some circumstances, they can maintain specificity. We speculate that the response to G, which provides energy as well as material, has features that are qualitatively different from the other responses. How the cell actually distinguishes between nutrients remains a mystery.

## Supplementary Material

Supporting Information
