# Supplementary material for: Glucose, Nitrogen, and Phosphate Repletion in Saccharomyces cerevisiae: Common Transcriptional Responses to Different Nutrient Signals
Source: G3 (Bethesda). 2012 Sep 1;2(9):1003–17. doi: 10.1534/g3.112.002808 (PMC3429914; doi:10.1534/g3.112.002808)
Supplement: Supporting Information [file supp_2_9_1003__index.html]

Supporting Information 

# Glucose, Nitrogen, and Phosphate Repletion in *Saccharomyces cerevisiae*: Common Transcriptional Responses to Different Nutrient Signals

## Supporting Information for Conway, Grunwald, and Heideman, 2012

**Files in this Data Supplement:**

- Supporting Information - Figure S1, File S1, and Tables S1 and S2 (PDF, 71 KB)
- Figure S1 - Expression levels of specific gene sets (PDF, 68 KB)
- File S1 - (.xls, 27.2 MB)
- Table S1 - (.xls, 291 KB)
- Table S2 - (.xls, 264 KB)
