# Supplementary material for: Glucose, Nitrogen, and Phosphate Repletion in Saccharomyces cerevisiae: Common Transcriptional Responses to Different Nutrient Signals
Source: G3 (Bethesda). 2012 Sep 1;2(9):1003–17. doi: 10.1534/g3.112.002808 (PMC3429914; doi:10.1534/g3.112.002808)
Supplement: Supporting Information [file supp_2.9.1003_FigureS1.pdf]

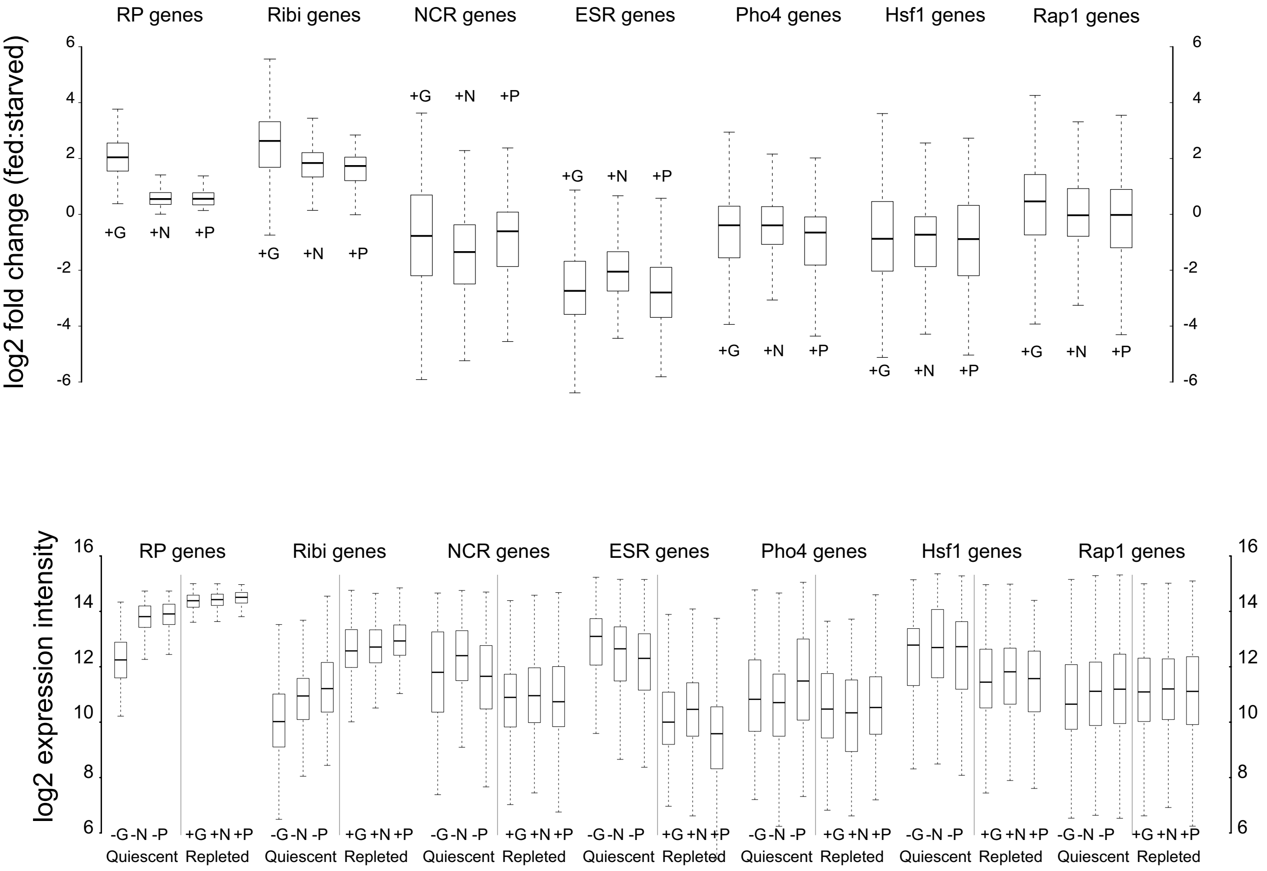

FIGURE 1 Expression levels of specific gene sets. The responses to the nutrient repletion experiment in Figure 2 were examined for specific gene sets: 137 RP and 230 Ribi genes Jorgensen *et al* (Jorgensen *et al.* 2004), 79 N catabolite repression (NCR) genes Scherens *et al* (Scherens *et al.* 2006), 272 environmental stress response genes (ESR) Gasch *et al* (Gasch *et al.* 2000), 95 Pho4 target genes Harbison *et al* (Harbison *et al.* 2004), 160 Hsf1 targets Hahn *et al* (Hahn *et al.* 2004), and 241 Rap1 targets Lieb *et al* 2001 (Lieb *et al.* 2001). A) Box plots showing the average log2 fold-change in expression caused by the indicated nutrient repletion, relative to the initial quiescent state. The black band in the box indicates the median, the upper box limit the 75th percentile, the lower box limit the 25th percentile and each whisker the minimum and maximum value within 1.5x of the interquartile range respectively. B) Box plots as above, except that the Y-axis shows averaged log2 signal intensity for the initial nutrient-limited cultures as well as the samples from the nutrient-repleted cultures. This shows how in some cases differences in the starting transcript levels altered the fold-change values as much as the transcript levels after repletion.
